# Increasing the uptake of vitamin D supplement use in Australian residential aged care facilities: results from the vitamin D implementation (ViDAus) study

**DOI:** 10.1186/s12877-020-01784-5

**Published:** 2020-10-06

**Authors:** Pippy Walker, Annette Kifley, Susan Kurrle, Ian D. Cameron

**Affiliations:** 1 Menzies Centre for Health Policy, University of Sydney, Charles Perkins Centre D17, Camperdown, NSW 2006 Australia; 2 John Walsh Centre for Rehabilitation Research, University of Sydney, Kolling Institute of Medical Research, Royal North Shore Hospital, St Leonards, NSW 2065 Australia; 3 NHMRC Cognitive Decline Partnership Centre, Hornsby Ku-ring-gai Health Service, Hornsby, NSW 2077 Australia

**Keywords:** Falls, Frail older adults, Preventive medicine, Residential facilities, Vitamin D

## Abstract

**Background:**

Adequate (≥800 IU/day) vitamin D supplement use in Australian residential aged care facilities (RACFs) is variable and non-optimal. The vitamin D implementation (ViDAus) study aimed to employ a range of strategies to support the uptake of this best practice in participating facilities. The aim of this paper is to report on facility level prevalence outcomes and factors associated with vitamin D supplement use.

**Methods:**

This trial followed a stepped wedge cluster, non-randomised design with 41 individual facilities serving as clusters pragmatically allocated into two wedges that commenced the intervention six months apart. This multifaceted, interdisciplinary knowledge translation intervention was led by a project officer, who worked with nominated champions at participating facilities to provide education and undertake quality improvement (QI) planning. Local barriers and responsive strategies were identified to engage stakeholders and promote widespread uptake of vitamin D supplement use.

**Results:**

This study found no significant difference in the change of vitamin D supplement use between the intervention (17 facilities with approx. 1500 residents) and control group (24 facilities with approx. 1900 residents) at six months (difference in prevalence change between groups was 1.10, 95% CI − 3.8 to 6.0, *p* = 0.6). The average overall facility change in adequate (≥800 IU/day) vitamin D supplement use over 12 months was 3.86% (95% CI 0.6 to 7.2, *p* = 0.02), which achieved a facility level average prevalence of 59.6%. The variation in uptake at 12 months ranged from 25 to 88% of residents at each facility. In terms of the types of strategies employed for implementation, there were no statistical differences between facilities that achieved a clinically meaningful improvement (≥10%) or a desired prevalence of vitamin D supplement use (80% of residents) compared to those that did not.

**Conclusions:**

This work confirms the complex nature of implementation of best practice in the RACF setting and indicates that more needs to be done to ensure best practice is translated into action. Whilst some strategies appeared to be associated with better outcomes, the statistical insignificance of these findings and the overall limited impact of the intervention suggests that the role of broader organisational and governmental support for implementation should be investigated further.

**Trial registration:**

Retrospectively registered (ANZCTR ID: ACTRN12616000782437).

## Background

Falls and fall-related injuries are a major cause of impaired quality of life in older people, particularly older people living in residential aged care facilities (RACFs) [1, 2]. The healthcare costs of falls are high, with trends in fall-related hospitalisations for RACF residents increasing [3]. Identifying and addressing the underlying causes of falls and fall-related injuries is therefore of critical importance, both to improve the health and wellbeing of older adults and to reduce health care costs.

There is strong evidence that residents of RACFs are at high risk of vitamin D deficiency, and that vitamin D deficiency is associated with falls in this group [1, 4–6]. Vitamin D deficiency is prevalent, with reports of between 68 and 86% of residents in Australian RACFs found to be deficient [5, 7, 8]. Furthermore, there is strong (Level I) evidence that vitamin D supplementation is both effective as a single intervention for reducing falls amongst older people in RACFs, and is cost-effective [1, 9]. This evidence is reflected in current best practice guidelines for the prevention of falls in RACFs and has more recently been confirmed as best practice by an expert panel [2, 10, 11].

Experts have recommended universal vitamin D supplementation as a population approach to falls and fracture prevention in RACFs [10]. Routine baseline and follow up blood tests in this population with high levels of vitamin D deficiency are discouraged as it is costly [10, 12], and although vitamin D supplements are not currently subsidised in Australia, the cost to residents is low [9, 12]. The need for universal vitamin D supplementation is supported by the knowledge that several barriers currently limit the use of outdoors spaces for adequate sun exposure by residents [7, 13, 14]. Secondly, dietary intake of vitamin D is low [8], and it is very unlikely that vitamin D fortified products are being provided given the low and reducing spend on food by RACFs in Australia [15].

Despite the evidence, vitamin D supplementation is not routinely prescribed in Australian RACFs. The prevalence of adequate vitamin D supplement use in Australia was recently found to vary considerably between individual facilities (15.9–85.0%), with a mean prevalence of 47.1% [16]. Although the uptake of vitamin D supplement use appears to be increasing, levels are still well below what would be considered clinically appropriate for a population-based intervention and requires further investigation [16, 17].

Implementation research is the ideal platform for this investigation, where strategies can be applied and tested to determine how such a change in practice can be achieved [18]. The aim of the ViDAus study was to test evidence-based strategies in the Australian context, after learning of recent successful national vitamin D implementation efforts in New Zealand [19–21]. It was hypothesised that by employing implementation strategies such as education, conducting audits, and facilitating system change to improve the timely identification of residents, an increase in the use of vitamin D supplements by residents could be achieved.

This paper reports on the effectiveness of this multifaceted, interdisciplinary knowledge translation intervention, with the primary aim being to increase the prevalence of adequate (≥800 IU/day) vitamin D supplement use among participating facilities. Secondary objectives were to evaluate the association of vitamin D supplement use with the adoption of selected implementation strategies, other medication variables (e.g. calcium supplement use, osteoporosis medication use and total number of prescribed oral medications) and fall rates.

## Methods

### Trial design

This was a pragmatic stepped wedge cluster non-randomised trial, which involved a 12-month intervention introduced at a six-month step. This allowed the second wedge to serve as the control group during the first six months (Table 1). The methods of this intervention have been reported in accordance with the template for intervention description and replication (TIDieR) checklist [22], and the TREND statement checklist of information to include when reporting a nonrandomised evaluation [23]. This paper provides a brief overview of the study participants, setting and intervention, with more detail available in a previously published paper on the study process outcomes [24].

**Table 1:** Stepped wedge trial design

Month	Wedge 1 Facilities	Wedge 2 Facilities
**0**	*Medication Chart Audit 1*	
**1–6**	*Intervention*	Control
**6**	*Medication Chart Audit 2*	
**7–12**	*Intervention*	*Intervention*
**12**	*Medication Chart Audit 3*	
**13–18**	Sustainment	*Intervention*
**18**	*Medication Chart Audit 4*	

### Participants

A convenience sample of aged care organisations were recruited through existing relationships with the research team, or from expression of interest after learning of the study from already recruited organisations. High level organisational managers nominated individual aged care facilities for recruitment into the trial. As obtaining consent or providing an opt-out approach for individuals would bias results, ethical approval to conduct this study without obtaining consent from individual participants was granted as per section 2.3 of the National Statement on Ethical Conduct in Human Research [25]. Written informed consent was sought from the manager of nominated aged care facilities prior to being allocated to wedge 1 or 2. Clusters were individual RACFs from four separate aged care organisations in New South Wales or South Australia. The only exclusion criteria applied were if facilities provided exclusively palliative care, had only bed bound residents, or had a prevalence of adequate (≥800 IU/day) vitamin D supplement use above 80%.

### Theoretical framework

The design of this intervention was based on the critical elements necessary for successful implementation outlined in the promoting action on research implementation in health services (PARIHS) framework [26]. This framework recognises the role of evidence (research, clinical experience, patient experience and local information), context (receptivity, culture, leadership and evaluation) and facilitation (purpose, role, skills and attributes) in the development of an implementation intervention and has been found to encapsulate the key factors perceived as important for change in the aged care setting [27–29].

### Interventions and implementation

To address these criteria for implementing evidence into practice, several implementation strategies were employed based on the available evidence of likely effect [30]. Strategies included identifying a local champion to drive implementation, the use of an expert opinion leader, dissemination of educational material, educational outreach visits, feedback on medication chart audits and locally facilitated quality improvement (QI) [30–33]. The detail of these interventions has been described elsewhere [24].

A project officer, who was a qualified accredited practising dietitian and accredited exercise physiologist was employed to coordinate the implementation of this multifaceted interdisciplinary knowledge translation intervention between December 2015 and June 2017. Key stakeholder groups identified were the servicing general practitioners (GPs) and pharmacists, residents, their relatives and facility staff members.

### Outcomes

The primary outcome measure of interest was the change in mean facility prevalence of adequate vitamin D supplement use, defined as equal to or greater than 800 IU/day. This was assessed at six, 12- and 18-month time points compared to baseline, with the six-month measure allowing for comparison between the intervention (wedge 1) and control (wedge 2) group (Table 1).

Secondary outcome measures include other medication chart variables to understand the prevalence of adequate (≥500 mg/day) calcium supplement use, osteoporosis medication use, total number of prescribed oral medications, demographic variables (age and sex) and falls rates. Period prevalence data were provided by servicing pharmacists in the form of de-identified reports from their patient medication chart database.

Falls rates were calculated by dividing the number of falls recorded over each six-month period, per 1000 occupied bed days over this time by facility. Aged care organisations provided de-identified falls reports following each six-month period. This was an open cohort, where outcomes were measured at a specific point in time to reflect the residents occupying aged care beds at that time (medication data) or during that previous six-month period (falls data).

The feasibility of this intervention has been reported on separately [24], however the association between the adoption of implementation strategies and the uptake of vitamin D supplement use achieved is explored here. Elements of the intervention that were to be adopted by stakeholders included the nomination of a key contact by participating facilities, their participation in education and the QI planning meeting, staff attendance at face-to-face education sessions, and the implementation of planned QI strategies.

Implementation strategies were evaluated by comparing facilities in two ways. Firstly, facilities that achieved a clinically meaningful improvement in vitamin D supplement use prevalence (> 10%) at 12 months were compared with those that did not, and secondly facilities that achieved a desired prevalence of vitamin D supplement use (≥80%) at 12 months were compared to those that did not.

### Study power

Sample size calculations indicated that with an anticipated recruitment of at least 32 care facilities including a total of 2240 residents, the study had more than 80% power to detect a difference of 25% versus 10% between the two wedges in mean change from baseline in the proportion of facility residents receiving vitamin D supplements at the six-month collection time point, with a significance level of 0.05. This effect size was based on a change from 50 to 75% in prevalence of adequate vitamin D supplementation following the intervention and allowed for up to a 10% increase in vitamin D supplement use among control facilities during the same period.

### Allocation of facilities

Individual participating RACFs were the unit of allocation. As participating facilities were managed and operated within regions, randomisation of sites was not possible as this would have left sites randomised into the second wedge open to contamination from sites in the first wedge that operate within the same organisational region. Previous implementation trials have experienced a similar issue [33, 34]. Randomising by region may have resulted in two uneven groups (the number of facilities in each region varied from 1 to 17), which was not a practical option for implementing the intervention, as it would not have been feasible to manage more than half of the participating facilities at one time. Facilities were allocated by the investigators to one of two wedges with other facilities within their operational region, though where possible achieving a spread of organisations and regional sites across the two groups.

### Blinding

The nature of this intervention did not allow for blinding of participating facilities, respective staff members and the project officer responsible for implementation. The primary outcome measure is objectively collected from medication charts.

### Statistical methods

The primary analysis compared change from baseline in the mean proportion of residents on adequate (≥800 IU/day) vitamin D supplementation at each facility between the intervention arm (first wedge) and the control arm (second wedge), at the six-month time point, using a direct analysis of change scores. To derive the denominator for the calculation of proportion of residents receiving supplementation, facilities were assumed to be operating at full capacity.

Secondary analyses assessed and compared changes from baseline at the 12-month and 18-month time points, and directly compared the mean post-intervention proportion of residents on adequate vitamin D supplementation at each facility between the two wedges at the six, 12- and 18-month time points, before and after adjusting for baseline using analysis of covariance (ANCOVA).

Fall rates per 1000 occupied bed days were calculated for each six-month period, and cumulatively at six, 12 and 18 months, from facility-level data on number of falls and bed capacity at each facility. Fall rates were compared between wedges, with a key focus on fall rates after 12 months. Negative binomial generalised linear models were used for unadjusted comparisons of fall rate per occupied bed days between wedges. Adjustment for baseline was then undertaken using longitudinal GEE models with autoregressive correlation. Differences between wedges in change from baseline of fall rates were assessed using Kruskal-Wallis tests.

Baseline characteristics were described at facility and/or individual level as appropriate, and baseline imbalances between wedges were assessed. Factors associated with vitamin D supplement usage were explored in individual-level analysis that accounted for clustering by facility.

Analyses were conducted using Statistical Analysis System (SAS) 9.4 and R 3.6.1.

## Results

### Participant flow

All 42 facilities that were recommended for recruitment met the inclusion criteria, however one withdrew prior to commencing the intervention due to the absence of a facility manager and resultant reduced capacity to participate in research during that time. Table 2 summarises the allocation of 41 participating sites (approx. 3400 residents) into two groups. The month that medication chart data were collected, and the proportion of the total bed capacity of each wedge included in each audit are summarised in Table 2, where usually at least 90% of total bed capacity was represented. The intervention was delivered between December 2015 and June 2017, with details of the implementation timeline reported on elsewhere [24].

**Table 2:** Facility allocation and medication chart audits as a proportion of total bed capacity

	**Wedge 1 Sites**	**Wedge 2 Sites**
Number of individual participating sites	17	24
Number of aged care organisations	2	4
Australian states represented	NSW	NSW & SA
Total reported bed capacity (n)	1544	1863
**Medication chart audits**	**n (% of total capacity)**	**n (% of total capacity)**
Audit 1 (December 2015)	1326 (86)	1670 (90)
Audit 2 (June 2016)	1415 (92)	1743 (94)
Audit 3 (December 2016)	1466 (95)	1732 (93)
Audit 4 (June 2017)	1418 (92)	1729 (93)
Average Occupancy	1406 (91)	1719 (92)

### Baseline data

Baseline characteristics of participating facilities are summarised in Table 3. Reported bed capacity ranged from 30 to 289 for participating facilities, with an average of 91 beds in the first wedge (range 40–289) and an average of 78 beds in the second wedge (range 30–155). Of these beds, 85% were in a major city, compared with 12% in an inner regional and 2% in an outer regional area. No beds were in a remote or very remote area.

**Table 3:** Baseline characteristics of participating facilities

Characteristic		Overall	Wedge 1	Wedge 2	Difference between groups (***p*** value)
Not-for-profit	Percent	100	100	100	–
Remoteness^a^	Percent aged care places in a major city	85.2	86.7	84.0	–
Bed capacity (excl. respite)	Mean	83.1	90.8	77.6	0.3
	(95% CI)	(68.6–97.5)	(60.0–121.6)	(63.8–91.4)	
	(range)	(30–289)			
Falls rate over 6 months prior to implementation^b^	Mean (Range)	5.6 (2.1–12.5)	4.9	6.1	0.092

*CI* Confidence Interval

^a^Remoteness Area [35]

^b^Falls rate per 1000 Occupied Bed Days

Baseline characteristics of study participants are summarised in Table 4. All facilities that expressed initial interest in participating in this study met the inclusion criteria of a prevalence of adequate vitamin D supplement use of less than 80%, with no significant difference in baseline prevalence between groups (Table 4). The only characteristic that was significantly different between the two groups was the average number of prescribed oral medications (*p* = 0.0025).

**Table 4:** Baseline characteristics of aged care residents

Characteristic		Overall	***Wedge 1***	***Wedge 2***	Difference between groups (***p*** value)
Age (years)	Mean	85.2	85.7	84.8	0.3
	(95% CI)	(84.2–86.2)	(84.7–86.6)	(83.1–86.4)	
	(individual range)	(48–107)			
Male sex	Percent	29.8	28.7	30.6	0.4
	(95% CI)	(27.1–32.4)	(25.4–32.0)	(26.7–35.0)	
	(% range)	(8.8–54.4)			
Adequate (≥800 IU/day) vitamin D supplement use	Percent	56.2	58.1	54.7	0.4
	(95% CI)	(51.4–61.0)	(53.0–63.1)	(47.1–62.3)	
	(% range)	(15.4–77.5)			
Adequate (≥500 mg/day) calcium supplement use	Percent	17.8	15.5	19.6	0.2
	(95% CI)	(13.8–21.8)	(12.2–18.9)	(13.3–26.0)	
	(% range)	(0–46.9)			
Osteoporosis medication use	Percent	15.3	14.5	15.9	0.4
	(95% CI)	(13.1–17.5)	(11.8–17.3)	(12.9–19.0)	
	(% range)	(3.8–40.0)			
Number of prescribed oral medications	Mean	10.9	9.8	11.8	**0.0025**
	(95% CI)	(10.2–11.7)	(8.8–10.9)	(11.1–12.5)	
	(individual range)	(0–43)			

*CI* Confidence Interval

### Medication chart audits

A significant increase in mean percentage of adequate vitamin D supplement use was seen overall during the study, from 55.0% at baseline to 58.8% at 12 months and 59.6% at 18 months, corresponding to a change from baseline of 3.86% at 12 months (95% CI 0.6 to 7.2, *p* = 0.02) and 4.58% at 18 months (95% CI 0.9 to 8.2, *p* = 0.015) (Table 5). There were no significance differences between the first wedge (intervention group) and the second wedge (control group) however in either the prevalence of adequate vitamin D supplement use, or in change from baseline in adequate vitamin D supplement use at any time point.

**Table 5:** Change in mean percentage of residents at each facility receiving adequate (≥800 IU/day) vitamin D supplementation at 6, 12 and 18 months compared to baseline

	Overall	Intervention Group (Wedge 1)	Control Group (Wedge 2)	Difference between groups (95% CI) p value	p value adjusted for baseline
Baseline Mean % (SD)	55.0 (16.0)	57.1 (14.5)	53.5 (17.2)	3.6 (−6.8, 14.0) *p* = 0.4	
6 Months Mean % (SD)	55.4 (13.9)	58.1 (13.4)	53.4 (14.2)	4.7 (−4.2, 13.6) *p* = 0.2	*p* = 0.4
12 Months Mean % (SD)	58.8 (14.2)	58.8 (13.4)	58.9 (13.8)	−0.1 (−9.3, 9.1) *p* = 0.9	*p* = 0.3
18 Months Mean % (SD)	59.6 (14.4)	60.5 (15.2)	58.9 (14.1)	1.6 (−9.3, 9.1) *p* = 0.6	*p* = 0.8
**Change from baseline**					
6 Month Mean % Change (95% CI) *p* value	0.40 (−2.2, 3.0) *p* = 0.7	1.05 *p* = 0.4	−0.05 *p* = 0.9	*1.10 (−3.8, 6.0)* *p = 0.6*	
12 Months Mean % Change (95% CI) *p* value	3.86 (0.6, 7.2) ***p = 0.02***	1.68 v = 0.3	5.41 *p* = 0.03	−3.73 (−10.3, 2.9) *p* = 0.2	
18 Month Mean % Change (95% CI) *p* value	4.58 (0.9, 8.2) ***p = 0.015***	3.42 *p* = 0.15	5.40 *p* = 0.053	−1.98 (−9.0, 5.1) *p* = 0.5	

*CI* Confidence Interval

The primary outcome for assessing the effectiveness of the intervention was a change from baseline in prevalence of adequate vitamin D supplement use at six months, and this differed by only 1.10% (95% CI − 3.8 to 6.0, *p* = 0.6) between the two wedges (Table 5). There was large variation in the individual facility change in adequate vitamin D supplement use over 12 months, from an increase of 32% to a reduction by 13%. The prevalence of residents at participating facilities prescribed an adequate dose of vitamin D achieved at 12 months also varied from between 25 to 88%.

### Ancillary analyses

#### Other medication variables

Adequate (≥500 mg/day) calcium supplement use, osteoporosis medication use, and the average number of prescribed oral medications did not change significantly over the course of the intervention. These variables also did not differ significantly between groups, except for average number of all prescribed oral medications, which was higher in facilities in the second wedge both at baseline and 18 months (*p* = 0.0025 and *p* = 0.004 respectively), and adequate calcium supplement use which was higher among facilities in the second wedge at 18 months (*p* = 0.0003).

After accounting for clustering by facility, individuals who were prescribed an adequate dose of a vitamin D supplement in audit one and audit four respectively were more likely to be prescribed an adequate (≥500 mg/day) dose of calcium (*p* < 0.001, *p* = 0.009), prescribed an osteoporosis medication (*p* < 0.0001, *p* < 0.0001), be female (*p* = 0.003, *p* = 0.0025) and more likely to be prescribed a higher number of total oral medications (p < 0.0001, p < 0.0001). Age was not found to be associated with adequate vitamin D supplement use in either audit one (*p* = 0.5) or audit four (*p* = 0.9).

There was a small but significant overall increase in falls over the intervention period, by 0.9 falls per 1000 occupied bed days compared to the pre-intervention period, but there were no significant differences in the rate of falls between groups at any period (Table 6). An exploratory analysis using generalised linear mixed models accounting for potential correlation between individuals within facilities and between time points within individuals found no significant association between the prevalence of adequate vitamin D supplement use and fall rates (*p* = 0.12).

**Table 6:** Rate of falls (number of falls per 1000 occupied bed days) at each facility

	Overall	Wedge 1	Wedge 2	Wedge 1 vs. Wedge 2	
	Mean (Range)	Mean (Range)	Mean (Range)	Unadjusted p value for raw difference^a^	Adjusted for baseline^a^
Audit 1: 6 months pre-baseline	5.6 (2.1–12.5)	4.9	6.1	*p* = 0.092	
Audit 2: Baseline to 6 months	6.4 (1.7–12.9)	5.4	7.1	*p* = 0.095	*p* = 0.4
Audit 3: 6 to 12 months	6.7 (1.7–12.9)	6.1	7.1	*p* = 0.3	*p* = 0.6
Audit 4: 12 to 18 months	7.0 (1.9–18.0)	5.8	7.7	*p* = 0.082	*p* = 0.7
Pre-intervention Period *(Wedge 1: Audit 1; Wedge 2: Audits 1 + 2)*	5.8 (2.1–12.7)	4.9 (2.7–9.2)	6.5 (2.1–12.7)		
Post Intervention Period *(Wedge 1: Audits 3 + 4; Wedge 2: Audit 4)*	6.7 (2.2–14.6)	5.8 (2.3–10.0)	7.4 (2.2–14.6)		
	Mean Change (signed rank test *p* value)			Kruskal-Wallis p value for difference between wedges	
Change Post Intervention vs. Pre-Intervention	0.9 (*p* = 0.015)	0.8 (*p* = 0.14)	0.9 (*p* = 0.05)	*p* = ~ 1	

^a^Negative binomial model used for fall rates per occupied bed days due to overdispersion in the Poisson model. Adjustment for baseline was achieved using a longitudinal GEE model with autoregressive correlation

#### Implementation strategies employed

The identified implementation strategies are presented in Table 7 to evaluate any association between uptake of strategies, and outcomes with respect to changes in adequate (≥800 IU/day) vitamin D supplement use prevalence, and overall prevalence achieved at 12 months. This exploratory analysis found no significant positive association between outcomes and the uptake of any implementation strategy, while the facilities without meaningful improvement in vitamin D supplement use (≤10%) were more likely to have emailed or made educational information available onsite to family members and residents (*p* = 0.04, *p* = 0.006 respectively).

**Table 7:** Characteristics and strategies implemented by facilities based on improvement in prevalence and total prevalence of adequate vitamin D supplement achieved at 12 months

	Clinically meaningful improvement in prevalence achieved (≥10%)?			Desired prevalence (≥80%) achieved?		
	Yes (***n*** = 10)	No (***n*** = 31)		Yes (***n*** = 2)	No (***n*** = 39)	
**Variable**	**Average for sites grouped by outcomes (%)**					
Baseline prevalence	47	58		68	54	
12-month prevalence	63	58		**84**	**58**	
Change in prevalence over 12 months	**16**	**0**		17	3	
**Implementation Strategy**	**Proportion that implemented each strategy (%)**					
			*p value*^*a*^			*p value*^*a*^
Key contact engagement (attendance at both education and QI planning meeting)	*70*	*55*	*0.4*	*100*	*56*	*0.5*
Provided additional staff education	10	3	0.4	0	5	~ 1
Provided resident education	50	52	~ 1	100	49	0.4
Provided family education	20	23	~ 1	50	21	0.3
Emailed families information	0	36	**0.04**	50	26	0.4
Made information available onsite	0	48	**0.006**	0	39	0.5
Added vitamin D to staff meeting agendas	30	23	0.6	50	23	0.4
Staff education uploaded into online portals	20	10	0.5	0	13	~ 1
Completed audit to identify residents for vitamin D	*70*	*42*	*0.15*	*100*	*46*	*0.2*
Implemented a local process to identify residents	60	36	0.2	100	39	0.16
General follow up with GPs	10	45	0.06	0	39	0.5
Follow up with GPs regarding individual residents	*60*	*26*	*0.06*	*100*	*31*	*0.11*
General follow up with pharmacists	30	29	~ 1	0	31	~ 1

^a^*p* value from Fishers exact test for association between adoption of implementation strategies and achievement of outcomes

## Discussion

This trial sought to increase the prevalence of adequate (≥800 IU/day) vitamin D supplement use among participating RACFs by employing a range of evidence based, locally tailored implementation strategies. The goal was to achieve an average post-intervention change of at least 25% (allowing for a 10% improvement in the control group due to practice improvements over time), given other large international efforts have achieved improvements from 22 to 50% (reaching a prevalence of between 58.2 and 66%) [20, 33, 36]. The overall facility level prevalence increased from 55.0 to 59.6% over an 18-month period with no clinically or statistically significant differences between wedges. Although a statistically significant overall change occurred at both 12 months (3.86, 95%CI 0.6, 7.2) and 18 months (4.58, 95%CI 0.9, 8.2), this change was clinically insignificant and was not the study’s primary endpoint.

This small change may be attributable to a higher baseline prevalence compared with other studies. These studies reported baselines of between 16 to 36% of residents prescribed vitamin D, which is much lower than the present study (55%) [20, 33, 36]. When comparing the change in prevalence between individual facilities, those that saw more than a 10% increase had a lower average baseline (47%), than those that did not (58%) (Table 7).

What is of great concern is the wide variation in uptake of vitamin D supplement use, with baselines ranging from 15.4 to 77.5% among participating facilities, which did not differ significantly between groups (*p* = 0.4). This is similar to the previous Australian audit which found a facility variation from between 15.9 to 85.0% [16]. Given this study and others have demonstrated that individual facility prevalence of over 80% can be achieved, there is still much room for improvement [17].

Given widespread vitamin D deficiency in RACFs and the knowledge that vitamin D supplement use can correct this deficiency and reduce the rate of falls, there is support for the implementation of vitamin D supplementation for all residents in RACFs [1, 5, 7, 8, 37, 38]. This is also supported by the finding that mobility has a non-linear relationship with falls risk, where Barker et al. [39] report even residents that are fully dependent are at risk of falling. Baeyens et al. [38] provides further justification of the relevance of vitamin D supplement use amongst residents having palliative care, who among other residents stand to gain additional benefits such as pain management and improved quality of life.

Despite this evidence, and other work backing a whole of population implementation approach [19, 36, 40], there are several factors that contradict this message. This includes recent guidelines that encourage vitamin D be deprescribed for residents who are at low falls risk [41], the focus placed on polypharmacy where many interpret this as a need to reduce the total number of medications [42], and other research placing a focus on residents at high risk, or receiving low levels of care in their analysis of implementation outcomes [21, 43, 44]. A population approach to prevention also goes against the training and traditional way medical practitioners work, in that assessing risk before providing treatment is usual practice [45]. Successful implementation efforts in New Zealand focused on a national approach with support from government, which may be a more effective approach to addressing these contradictions [19–21].

The association of vitamin D supplement use with age and sex in this study contradicted a recent audit in Tasmania, where older age (*p* = 0.003), but not sex (*p* = 0.398) was associated with vitamin D supplement use [46]. This suggests that variation in outcomes is the result of a knowledge translation gap, rather than the cohort and respective care needs of residents.

Key contact engagement, conducting an internal audit to identify residents not prescribed an adequate (≥800 IU/day) dose of vitamin D, implementing local processes to identify residents suitable for vitamin D, and following up with GPs to refer identified residents were the strategies employed by a higher proportion of facilities that saw favourable outcomes compared to those that did not, however the association between the adoption of these strategies and achievement of outcomes at 12 months was not significant (Table 7).

Internal facilitation, audit and feedback and prescriber engagement have all previously been used successfully elsewhere for the implementation of vitamin D supplements [17, 32, 43, 47], and have been recognised in the literature more broadly as effective strategies [30, 48, 49]. It was less clear whether efforts to raise stakeholder awareness were effective, however given the feasibility, accepted importance and evidence for educational outreach as part of a multifaceted strategy, future efforts should not overlook its contribution [30, 50, 51].

Also, worth noting is the worryingly low level of adequate (≥500 mg/day) calcium supplement and osteoporosis medication use (Table 4). This is much lower than rates in Canada [33], and although not the aim did not change throughout the intervention. Given the association between vitamin D and calcium supplement use found in this and a recent audit [16], including more of a focus on providing guidance collectively on osteoporosis and fracture prevention and management may have contributed to a broader uptake of vitamin D.

The only medication variable to differ significantly between groups was the average number of prescribed oral medications (*p* = 0.0025), where the second wedge facilities had a higher average (12 medications) than both the first wedge and previous reports of around ten medications per resident [52, 53]. We found that a higher number of prescribed oral medications was associated with vitamin D supplement use (*p* < 0.0001) and by most definitions in the literature polypharmacy is present among this cohort of Australian RACF residents [42].

It was beyond the scope of this study to investigate the association of vitamin D supplement use and falls. Since we did not measure serum vitamin D, nor do we know whether residents who had falls were prescribed vitamin D, the effect of this intervention on falls cannot be ascertained [54]. Collecting and reporting on falls was included at the request of and expectation from participating organisations, as it was felt that this would be expected by stakeholders given the overall aim of prescribing vitamin D is to reduce falls, however the evidence on this effect is already clear and was not the primary focus of this study [1]. As expected, this trial had no impact on, and vitamin D supplement use prevalence was not found to be associated with the rate of falls (Table 6).

There are methodological limitations worth noting. Critically, the six-month comparison (primary outcome measure) only reflected the impact of educational outreach prior to implementation of other QI strategies, which are known to be essential for successful implementation [27, 55, 56]. It is for this reason that the interpretation of results has focused more on the change in prevalence from baseline, rather than comparing the intervention (wedge 1) and control group (wedge 2), as the intervention group had not implemented most of the intervention when this comparison was made.

As the timeline was set for this project based on funding arrangements, the intervention period was limited to 12 months, and control group comparison to six months. This is less than other successful implementation efforts, for instance with Campbell and Robertson [19] evaluating a three year roll out and Ward et al. [34] suggesting that their 17-month intervention was too short. This is evidenced here, where improvements continued to be seen between 12 and 18 months, suggesting that there may be some delay in implementation (Table 5).

As the sample of participating aged care organisations was a convenience sample, the findings may not be entirely generalisable across the Australian aged care setting. For example, this sample involved only facilities in the not-for-profit sector, which only made up 57% of operational aged care places in Australia in 2014 [57]. Similarly, there were more beds in major cities (85% vs. 68%), and fewer beds in inner regional (12% vs. 21%) and outer regional (2% vs. 9%) areas. Although no beds were from remote or very remote areas, only 0.9 and 0.5% of Australian aged care places are remote and very remote respectively [57]. The authors also acknowledge that this study did not consider variation in individual participating facility fees, or the socioeconomic status of residents.

Although the representation of Australian states differed between groups (no South Australian sites allocated to wedge 1), the total study cohort did represent 4 and 5% of operational aged care places in NSW and SA respectively [57]. Average age and proportion of male residents was also not different from previous reports of an average age of 84.5 years and 31% of residents being male [57]. Similarly, baseline fall rates (2 to 13 falls per 1000 OBD) were like previous reports of variation between 4 to 10 falls per 1000 OBD dependent on resident case mix [2].

Medication chart data were collected at arm’s length from the researchers. As pharmacists used a variety of different methods to provide the requested information, and often had different staff providing subsequent rounds of data, consistency and therefore reliability of the audit results cannot be verified. This study has also not considered differences in vitamin D formulation (e.g. vitamin D2 vs. vitamin D3), routes or frequency of administration (e.g. tablet, oral spray, injection), or issues with medication adherence.

The number of residents in each dataset was usually less than the reported total bed capacity. Although this may be representative of bed occupancy at the time, it became apparent that residents who self-administer their medications may not have vitamin D listed on their medication chart if the packing pharmacist does not provide this. Reports from some dispensing computer programs also only included residents that were current on the system at the time the report was produced. The impact of any error is likely to be very small however since the proportion of residents included in the audits was only 1% lower than bed occupancy in Australian aged care (92% vs. 93%) [57].

## Conclusions

Overall, this multifaceted interdisciplinary knowledge translation intervention failed to achieve a clinically or statistically significant improvement, however it achieved a prevalence of vitamin D supplement use comparable to other international efforts. The variability between individual facilities substantiates the complex nature of implementation in the residential aged care setting due to the interplay of numerous stakeholder and related contextual factors.

Given the wide variation in implementation success, it is difficult to draw certainty on what would work in any given aged care facility. The inconclusive evidence of association between implementation strategies employed and vitamin D supplement use outcomes suggests that it is unlikely that widespread improvements will be achieved until a consistent national message is delivered to overcome existing contradictions in prescribing guidelines and practices for residents in RACFs.

## Data Availability

The datasets generated and analysed during the current study are available from the corresponding author on reasonable request.

## Abbreviations

ANZCTR ID: Australian New Zealand Clinical Trials Registry Trial Identification
CI: Confidence Interval
GP: General Practitioner
IU: International Units
OBD: Occupied bed days
QI: Quality Improvement
RACF: Residential aged care facility
ViDAus: Vitamin D Implementation Study
